# Supplementary material for: Functional proteomic atlas of HIV infection in primary human CD4+ T cells
Source: eLife. 2019 Mar 12;8:e41431. doi: 10.7554/eLife.41431 (PMC6414203; doi:10.7554/eLife.41431)
Supplement: Supplementary file 1. [file elife-41431-supp1.docx]

**Supplementary file 1. gBlock and HIV-AFMACS sequences**

gBlock #1

tgtttatccatttcagaattgggtgtcgacatagcagaataggcgttactcgacagaggagagcaagaaatggagccagtagatcctagactagagccctggaagcatccaggaagtcagcctaaaactgcttgtaccaattgctattgtaaaaagtgttgctttcattgccaagtttgtttcatgacaaaagccttaggcatctcctatggcaggaagaagcggagacagcgacgaagagctcatcagaacagtcagactcatcaagcttctctatcaaagcagtaagtagtacatgtaatgcaacctataatagtagcaatagtagcattagtagtagcaataataatagcaatagttgtgtggtccatagtaatcatagaatataggaaaatattaagacaaagaaaaatagacaggttaattgatagactaatagaaagagcagaagacagtggcaatgagagtgaaggagaagtatcagcacttgtggagatgggggtggaaatggggcaccatgctccttgggatattgatgatctgtagtgctacagaaaaattgtgggtcacagtctattatggggtacctgtcgcggccgcagggtcagggatggacgaaaagacaaccggatggcgaggaggacacgtggtcgagggactggcaggagagctggaacagctgcgggctagactggaacaccatcctcagggacagcgagagcccggaagtggaaaggaagcctgccccacagggctgtacactcattctggagaatgctgtaaagcttgtaacctgggagagggagtggcacagccatgcggagccaatcagactgtgtgcgagccttgtctggactccgtcacattctctgatgtggtcagtgccacagaaccttgcaagccatgtactgagtgcgtgggcctgcagtctatgagtgctccttgtgtggaggctgacgatgcagtctgccggtgtgcatacggatactatcaggacgagactaccggcagatgtgaagcttgcagggtgtgtgaggcaggctcagggctggtctttagctgccaggataaacagaacaccgtgtgcgaggaatgtcctgacgggacatatagcgatgaggccaatcacgtggacccctgcctgccttgtactgtgtgcgaggataccgaaaggcagctgcgcgaatgtaccagatgggcagacgccgagtgcgaggaaatcccagggcgatggattactcggtccaccccccctgaaggatcagacagcaccgcaccatctacacaggagccagaagcaccaccagagcaggatctgatcgcctccaccgtggctggcgtggtcacaactgtcatggggagctctcagccagtggtcacccggggcaccacagataacctgattcccgtgtattgctccatcctggcagccgtcgtcgtcggactggtggcatacatcgccttcaagcggtgagctagccgtaacgatgttcatcaaatattactgggctgctat

gBlock #2

tcaggaactaaagaatagtgctgttaacttgctcaatgccacagccatagcagtagctgaggggacagatagggttatagaagtattacaagcagcttatagagctattcgccacatacctagaagaataagacagggcttggaaaggattttgctataagatgggggggaaatggtctaagtcttccgtgataggctggcccgctgttcgagaaaggatgcgccgcgccgaaccagcggccgatggcgtaggtgccgtatctagggaccttgagaagcacggtgccataacgtcttctaacaccgcagccaacaatgcggcatgtgcgtggttggaggcacaggaagaggaggaggtaggattcccggtaacgccacaagtacccttgcgccctatgacctacaaggccgccgtggacctgagccatttcctgaaggagaaaggtggcttggaagggcttatccactcccaaagacgccaggatattcttgacctttggatctaccacacacagggttactttcccgactggcaaaattatacgcccggtcccggtgtacggtatcctcttacttttgggtggtgctataaactcgtgccggtggagccagataaggtagaggaggccaataaaggtgagaacacaagtttgcttcaccccgtttcccttcatggtatggacgacccagaacgggaagttcttgaatggcggttcgatagcaggttggcatttcatcatgtcgcacgggagcttcaccccgagtactttaaaaattgtgcggccgcaggaagcggagctactaacttcagcctgctgaagcaggctggagacgtggaggagaaccctggacctatgggctggtcatgtatcattctgtttctggtcgcaaccgcaactggagtgcattcacaggtgcagctcagcgctgggtcagggatggacgaaaagacaaccggatggcgaggaggacacgtggtcgagggactggcaggagagctggaacagctgcgggctagactggaacaccatcctcagggacagcgagagcccggaagtggagcgatcgcgaaggaagcctgccccacagggctgtacactcattctggagaatgctgtaaagcttgtaacctgggagagggagtggcacagccatgcggagccaatcagactgtgtgcgagccttgtctggactccgtcacattctctgatgtggtcagtgccacagaaccttgcaagccatgtactgagtgcgtgggcctgcagtctatgagtgctccttgtgtggaggctgacgatgcagtctgccggtgtgcatacggatactatcaggacgagactaccggcagatgtgaagcttgcagggtgtgtgaggcaggctcagggctggtctttagctgccaggataaacagaacaccgtgtgcgaggaatgtcctgacgggacatatagcgatgaggccaatcacgtggacccctgcctgccttgtactgtgtgcgaggataccgaaaggcagctgcgcgaatgtaccagatgggcagacgccgagtgcgaggaaatcccagggcgatggattactcggtccaccccccctgaaggatcagacagcaccgcaccatctacacaggagccagaagcaccaccagagcaggatctgatcgcctccaccgtggctggcgtggtcacaactgtcatggggagctctcagccagtggtcacccggggcaccacagataacctgattcccgtgtattgctccatcctggcagccgtcgtcgtcggactggtggcatacatcgccttcaagcggtgactcgagacctagaaaaacatggagcaatca

gBlock #3

ggagtcaggaactaaagaatagtgctgttaacttgctcaatgccacagccatagcagtagctgaggggacagatagggttatagaagtattacaagcagcttatagagctattcgccacatacctagaagaataagacagggcttggaaaggattttgctataagatgggggggaaatggtctaagtcttccgtgataggctggcccgctgttcgagaaaggatgcgccgcgccgaaccagcggccgatggcgtaggtgccgtatctagggaccttgagaagcacggtgccataacgtcttctaacaccgcagccaacaatgcggcatgtgcgtggttggaggcacaggaagaggaggaggtaggattcccggtaacgccacaagtacccttgcgccctatgacctacaaggccgccgtggacctgagccatttcctgaaggagaaaggtggcttggaagggcttatccactcccaaagacgccaggatattcttgacctttggatctaccacacacagggttactttcccgactggcaaaattatacgcccggtcccggtgtacggtatcctcttacttttgggtggtgctataaactcgtgccggtggagccagataaggtagaggaggccaataaaggtgagaacacaagtttgcttcaccccgtttcccttcatggtatggacgacccagaacgggaagttcttgaatggcggttcgatagcaggttggcatttcatcatgtcgcacgggagcttcaccccgagtactttaaaaattgttgatctagacgcccccccctaacgttactggccgaagccgcttggaataaggccggtgtgcgtttgtctatatgttattttccaccatattgccgtcttttggcaatgtgagggcccggaaacctggccctgtcttcttgacgagcattcctaggggtctttcccctctcgccaaaggaatgcaaggtctgttgaatgtcgtgaaggaagcagttcctctggaagcttcttgaagacaaacaacgtctgtagcgaccctttgcaggcagcggaaccccccacctggcgacaggtgcctctgcggccaaaagccacgtgtataagatacacctgcaaaggcggcacaaccccagtgccacgttgtgagttggatagttgtggaaagagtcaaatggctctcctcaagcgtattcaacaaggggctgaaggatgcccagaaggtaccccattgtatgggatctgatctggggcctcggtgcacatgctttacatgtgtttagtcgaggttaaaaaacgtctaggccccccgaaccacggggacgtggttttcctttgaaaaacacgatgataatatgggctggtcatgtatcattctgtttctggtcgcaaccgcaactggagtgcattcacaggtgcagctcagcgctgggtcagggatggacgaaaagacaaccggatggcgaggaggacacgtggtcgagggactggcaggagagctggaacagctgcgggctagactggaacaccatcctcagggacagcgagagcccggaagtggagcgatcgcgaaggaagcctgccccacagggctgtacactcattctggagaatgctgtaaagcttgtaacctgggagagggagtggcacagccatgcggagccaatcagactgtgtgcgagccttgtctggactccgtcacattctctgatgtggtcagtgccacagaaccttgcaagccatgtactgagtgcgtgggcctgcagtctatgagtgctccttgtgtggaggctgacgatgcagtctgccggtgtgcatacggatactatcaggacgagactaccggcagatgtgaagcttgcagggtgtgtgaggcaggctcagggctggtctttagctgccaggataaacagaacaccgtgtgcgaggaatgtcctgacgggacatatagcgatgaggccaatcacgtggacccctgcctgccttgtactgtgtgcgaggataccgaaaggcagctgcgcgaatgtaccagatgggcagacgccgagtgcgaggaaatcccagggcgatggattactcggtccaccccccctgaaggatcagacagcaccgcaccatctacacaggagccagaagcaccaccagagcaggatctgatcgcctccaccgtggctggcgtggtcacaactgtcatggggagctctcagccagtggtcacccggggcaccacagataacctgattcccgtgtattgctccatcctggcagccgtcgtcgtcggactggtggcatacatcgccttcaagcggtgactcgagacctagaaaaacatggagcaatcacaag

gBlock #4

tgtttatccatttcagaattgggtgtcgacatagcagaataggcgttactcgacagaggagagcaagaaatggagccagtagatcctagactagagccctggaagcatccaggaagtcagcctaaaactgcttgtaccaattgctattgtaaaaagtgttgctttcattgccaagtttgtttcatgacaaaagccttaggcatctcctatggcaggaagaagcggagacagcgacgaagagctcatcagaacagtcagactcatcaagcttctctatcaaagcagtaagtagtacatgtaatgcaacctataatagtagcaatagtagcattagtagtagcaataataatagcaatagttgtgtggtccatagtaatcatagaatataggaaaatattaagacaaagaaaaatagacaggttaattgatagactaatagaaagagcagaagacagtggcaacgagagtgaaggagaagtatcagcacttgtggagatgggggtggaaatggggcaccacgctccttgggatattgacgatctgtaggctagccgtaacgatgttcatcaaatattactgggctgctat

gBlock #5

atacatcgccttcaagcggtgactcgagtagccactttttaaaagaaaaggggggactggaagggctaattcactcccaaagaagacaagatatcccatccggagtacttcaagaactgctgacatcgagcttgctacaagggactttccgctggggactttccagggaggcgtggcctgggcgggactggggagtggcgagccctcagatgctgcatataagcagctgctttttgcctgtactgggtctctctggttagaccagatctgagcctgggagctctctggctaactagggaacccactgcttaagcctcaataaagcttgccttgagtgcttcaagtagtgtgtgcccgtctgttgtgtgactctggtaactagagatccctcagacccttttagtcagtgtggaaaatctctagcacccaggaggtagaggttgcagtgagccaagatcgcgccactgcattccagcctgggcaagaaaacaagactgtctaaaataataataataagttaagggtattaaatatatttatacatggaggtcataaaaatatatatatttgggctgggcgcagtggctcacacctgcgcccggccctttgggaggccgaggcaggtggatcacctgagtttgggagttccagaccagcctgaccaacatggagaaaccccttctctgtgtatttttagtagattttattttatgtgtattttattcacaggtatttctggaaaactgaaactgtttttcctctactctgataccacaagaatcatcagcacagaggaagacttctgtgatcaaatgtggtgggagagggaggttttcaccagcacatgagcagtcagttctgccgcagactcggcgggtgtccttcggttcagttccaacaccgcctgcctggagagaggtcagaccacagggtgagggctcagtccccaagacataaacacccaagacataaacaccccaggtccaccccgcctgctgcccaggcagagccgattcaccaagacgggaattaggatagagaaagagtaagtcacacagagccggctgtgcgggagaacggagttcta

HIV-AFMACS (pNL4-3-ΔEnv-Nef-P2A-SBP-ΔLNGFR)

tggaagggctaatttggtcccaaaaaagacaagagatccttgatctgtggatctaccacacacaaggctacttccctgattggcagaactacacaccagggccagggatcagatatccactgacctttggatggtgcttcaagttagtaccagttgaaccagagcaagtagaagaggccaatgaaggagagaacaacagcttgttacaccctatgagccagcatgggatggaggacccggagggagaagtattagtgtggaagtttgacagcctcctagcatttcgtcacatggcccgagagctgcatccggagtactacaaagactgctgacatcgagctttctacaagggactttccgctggggactttccagggaggtgtggcctgggcgggactggggagtggcgagccctcagatgctacatataagcagctgctttttgcctgtactgggtctctctggttagaccagatctgagcctgggagctctctggctaactagggaacccactgcttaagcctcaataaagcttgccttgagtgctcaaagtagtgtgtgcccgtctgttgtgtgactctggtaactagagatccctcagacccttttagtcagtgtggaaaatctctagcagtggcgcccgaacagggacttgaaagcgaaagtaaagccagaggagatctctcgacgcaggactcggcttgctgaagcgcgcacggcaagaggcgaggggcggcgactggtgagtacgccaaaaattttgactagcggaggctagaaggagagagatgggtgcgagagcgtcggtattaagcgggggagaattagataaatgggaaaaaattcggttaaggccagggggaaagaaacaatataaactaaaacatatagtatgggcaagcagggagctagaacgattcgcagttaatcctggccttttagagacatcagaaggctgtagacaaatactgggacagctacaaccatcccttcagacaggatcagaagaacttagatcattatataatacaatagcagtcctctattgtgtgcatcaaaggatagatgtaaaagacaccaaggaagccttagataagatagaggaagagcaaaacaaaagtaagaaaaaggcacagcaagcagcagctgacacaggaaacaacagccaggtcagccaaaattaccctatagtgcagaacctccaggggcaaatggtacatcaggccatatcacctagaactttaaatgcatgggtaaaagtagtagaagagaaggctttcagcccagaagtaatacccatgttttcagcattatcagaaggagccaccccacaagatttaaataccatgctaaacacagtggggggacatcaagcagccatgcaaatgttaaaagagaccatcaatgaggaagctgcagaatgggatagattgcatccagtgcatgcagggcctattgcaccaggccagatgagagaaccaaggggaagtgacatagcaggaactactagtacccttcaggaacaaataggatggatgacacataatccacctatcccagtaggagaaatctataaaagatggataatcctgggattaaataaaatagtaagaatgtatagccctaccagcattctggacataagacaaggaccaaaggaaccctttagagactatgtagaccgattctataaaactctaagagccgagcaagcttcacaagaggtaaaaaattggatgacagaaaccttgttggtccaaaatgcgaacccagattgtaagactattttaaaagcattgggaccaggagcgacactagaagaaatgatgacagcatgtcagggagtggggggacccggccataaagcaagagttttggctgaagcaatgagccaagtaacaaatccagctaccataatgatacagaaaggcaattttaggaaccaaagaaagactgttaagtgtttcaattgtggcaaagaagggcacatagccaaaaattgcagggcccctaggaaaaagggctgttggaaatgtggaaaggaaggacaccaaatgaaagattgtactgagagacaggctaattttttagggaagatctggccttcccacaagggaaggccagggaattttcttcagagcagaccagagccaacagccccaccagaagagagcttcaggtttggggaagagacaacaactccctctcagaagcaggagccgatagacaaggaactgtatcctttagcttccctcagatcactctttggcagcgacccctcgtcacaataaagataggggggcaattaaaggaagctctattagatacaggagcagatgatacagtattagaagaaatgaatttgccaggaagatggaaaccaaaaatgatagggggaattggaggttttatcaaagtaagacagtatgatcagatactcatagaaatctgcggacataaagctataggtacagtattagtaggacctacacctgtcaacataattggaagaaatctgttgactcagattggctgcactttaaattttcccattagtcctattgagactgtaccagtaaaattaaagccaggaatggatggcccaaaagttaaacaatggccattgacagaagaaaaaataaaagcattagtagaaatttgtacagaaatggaaaaggaaggaaaaatttcaaaaattgggcctgaaaatccatacaatactccagtatttgccataaagaaaaaagacagtactaaatggagaaaattagtagatttcagagaacttaataagagaactcaagatttctgggaagttcaattaggaataccacatcctgcagggttaaaacagaaaaaatcagtaacagtactggatgtgggcgatgcatatttttcagttcccttagataaagacttcaggaagtatactgcatttaccatacctagtataaacaatgagacaccagggattagatatcagtacaatgtgcttccacagggatggaaaggatcaccagcaatattccagtgtagcatgacaaaaatcttagagccttttagaaaacaaaatccagacatagtcatctatcaatacatggatgatttgtatgtaggatctgacttagaaatagggcagcatagaacaaaaatagaggaactgagacaacatctgttgaggtggggatttaccacaccagacaaaaaacatcagaaagaacctccattcctttggatgggttatgaactccatcctgataaatggacagtacagcctatagtgctgccagaaaaggacagctggactgtcaatgacatacagaaattagtgggaaaattgaattgggcaagtcagatttatgcagggattaaagtaaggcaattatgtaaacttcttaggggaaccaaagcactaacagaagtagtaccactaacagaagaagcagagctagaactggcagaaaacagggagattctaaaagaaccggtacatggagtgtattatgacccatcaaaagacttaatagcagaaatacagaagcaggggcaaggccaatggacatatcaaatttatcaagagccatttaaaaatctgaaaacaggaaagtatgcaagaatgaagggtgcccacactaatgatgtgaaacaattaacagaggcagtacaaaaaatagccacagaaagcatagtaatatggggaaagactcctaaatttaaattacccatacaaaaggaaacatgggaagcatggtggacagagtattggcaagccacctggattcctgagtgggagtttgtcaatacccctcccttagtgaagttatggtaccagttagagaaagaacccataataggagcagaaactttctatgtagatggggcagccaatagggaaactaaattaggaaaagcaggatatgtaactgacagaggaagacaaaaagttgtccccctaacggacacaacaaatcagaagactgagttacaagcaattcatctagctttgcaggattcgggattagaagtaaacatagtgacagactcacaatatgcattgggaatcattcaagcacaaccagataagagtgaatcagagttagtcagtcaaataatagagcagttaataaaaaaggaaaaagtctacctggcatgggtaccagcacacaaaggaattggaggaaatgaacaagtagataaattggtcagtgctggaatcaggaaagtactatttttagatggaatagataaggcccaagaagaacatgagaaatatcacagtaattggagagcaatggctagtgattttaacctaccacctgtagtagcaaaagaaatagtagccagctgtgataaatgtcagctaaaaggggaagccatgcatggacaagtagactgtagcccaggaatatggcagctagattgtacacatttagaaggaaaagttatcttggtagcagttcatgtagccagtggatatatagaagcagaagtaattccagcagagacagggcaagaaacagcatacttcctcttaaaattagcaggaagatggccagtaaaaacagtacatacagacaatggcagcaatttcaccagtactacagttaaggccgcctgttggtgggcggggatcaagcaggaatttggcattccctacaatccccaaagtcaaggagtaatagaatctatgaataaagaattaaagaaaattataggacaggtaagagatcaggctgaacatcttaagacagcagtacaaatggcagtattcatccacaattttaaaagaaaaggggggattggggggtacagtgcaggggaaagaatagtagacataatagcaacagacatacaaactaaagaattacaaaaacaaattacaaaaattcaaaattttcgggtttattacagggacagcagagatccagtttggaaaggaccagcaaagctcctctggaaaggtgaaggggcagtagtaatacaagataatagtgacataaaagtagtgccaagaagaaaagcaaagatcatcagggattatggaaaacagatggcaggtgatgattgtgtggcaagtagacaggatgaggattaacacatggaaaagattagtaaaacaccatatgtatatttcaaggaaagctaaggactggttttatagacatcactatgaaagtactaatccaaaaataagttcagaagtacacatcccactaggggatgctaaattagtaataacaacatattggggtctgcatacaggagaaagagactggcatttgggtcagggagtctccatagaatggaggaaaaagagatatagcacacaagtagaccctgacctagcagaccaactaattcatctgcactattttgattgtttttcagaatctgctataagaaataccatattaggacgtatagttagtcctaggtgtgaatatcaagcaggacataacaaggtaggatctctacagtacttggcactagcagcattaataaaaccaaaacagataaagccacctttgcctagtgttaggaaactgacagaggacagatggaacaagccccagaagaccaagggccacagagggagccatacaatgaatggacactagagcttttagaggaacttaagagtgaagctgttagacattttcctaggatatggctccataacttaggacaacatatctatgaaacttacggggatacttgggcaggagtggaagccataataagaattctgcaacaactgctgtttatccatttcagaattgggtgtcgacatagcagaataggcgttactcgacagaggagagcaagaaatggagccagtagatcctagactagagccctggaagcatccaggaagtcagcctaaaactgcttgtaccaattgctattgtaaaaagtgttgctttcattgccaagtttgtttcatgacaaaagccttaggcatctcctatggcaggaagaagcggagacagcgacgaagagctcatcagaacagtcagactcatcaagcttctctatcaaagcagtaagtagtacatgtaatgcaacctataatagtagcaatagtagcattagtagtagcaataataatagcaatagttgtgtggtccatagtaatcatagaatataggaaaatattaagacaaagaaaaatagacaggttaattgatagactaatagaaagagcagaagacagtggcaacgagagtgaaggagaagtatcagcacttgtggagatgggggtggaaatggggcaccacgctccttgggatattgacgatctgtaggctagccgtaacgatgttcatcaaatattactgggctgctattaacaagagatggtggtaataacaacaatgggtccgagatcttcagacctggaggaggcgatatgagggacaattggagaagtgaattatataaatataaagtagtaaaaattgaaccattaggagtagcacccaccaaggcaaagagaagagtggtgcagagagaaaaaagagcagtgggaataggagctttgttccttgggttcttgggagcagcaggaagcactatgggcgcagcgtcaatgacgctgacggtacaggccagacaattattgtctgatatagtgcagcagcagaacaatttgctgagggctattgaggcgcaacagcatctgttgcaactcacagtctggggcatcaaacagctccaggcaagaatcctggctgtggaaagatacctaaaggatcaacagctcctggggatttggggttgctctggaaaactcatttgcaccactgctgtgccttggaatgctagttggagtaataaatctctggaacagatttggaataacatgacctggatggagtgggacagagaaattaacaattacacaagcttaatacactccttaattgaagaatcgcaaaaccagcaagaaaagaatgaacaagaattattggaattagataaatgggcaagtttgtggaattggtttaacataacaaattggctgtggtatataaaattattcataatgatagtaggaggcttggtaggtttaagaatagtttttgctgtactttctatagtgaatagagttaggcagggatattcaccattatcgtttcagacccacctcccaatcccgaggggacccgacaggcccgaaggaatagaagaagaaggtggagagagagacagagacagatccattcgattagtgaacggatccttagcacttatctgggacgatctgcggagcctgtgcctcttcagctaccaccgcttgagagacttactcttgattgtaacgaggattgtggaacttctgggacgcagggggtgggaagccctcaaatattggtggaatctcctacagtattggagtcaggaactaaagaatagtgctgttaacttgctcaatgccacagccatagcagtagctgaggggacagatagggttatagaagtattacaagcagcttatagagctattcgccacatacctagaagaataagacagggcttggaaaggattttgctataagatgggggggaaatggtctaagtcttccgtgataggctggcccgctgttcgagaaaggatgcgccgcgccgaaccagcggccgatggcgtaggtgccgtatctagggaccttgagaagcacggtgccataacgtcttctaacaccgcagccaacaatgcggcatgtgcgtggttggaggcacaggaagaggaggaggtaggattcccggtaacgccacaagtacccttgcgccctatgacctacaaggccgccgtggacctgagccatttcctgaaggagaaaggtggcttggaagggcttatccactcccaaagacgccaggatattcttgacctttggatctaccacacacagggttactttcccgactggcaaaattatacgcccggtcccggtgtacggtatcctcttacttttgggtggtgctataaactcgtgccggtggagccagataaggtagaggaggccaataaaggtgagaacacaagtttgcttcaccccgtttcccttcatggtatggacgacccagaacgggaagttcttgaatggcggttcgatagcaggttggcatttcatcatgtcgcacgggagcttcaccccgagtactttaaaaattgtgcggccgcaggaagcggagctactaacttcagcctgctgaagcaggctggagacgtggaggagaaccctggacctatgggctggtcatgtatcattctgtttctggtcgcaaccgcaactggagtgcattcacaggtgcagctcagcgctgggtcagggatggacgaaaagacaaccggatggcgaggaggacacgtggtcgagggactggcaggagagctggaacagctgcgggctagactggaacaccatcctcagggacagcgagagcccggaagtggagcgatcgcgaaggaagcctgccccacagggctgtacactcattctggagaatgctgtaaagcttgtaacctgggagagggagtggcacagccatgcggagccaatcagactgtgtgcgagccttgtctggactccgtcacattctctgatgtggtcagtgccacagaaccttgcaagccatgtactgagtgcgtgggcctgcagtctatgagtgctccttgtgtggaggctgacgatgcagtctgccggtgtgcatacggatactatcaggacgagactaccggcagatgtgaagcttgcagggtgtgtgaggcaggctcagggctggtctttagctgccaggataaacagaacaccgtgtgcgaggaatgtcctgacgggacatatagcgatgaggccaatcacgtggacccctgcctgccttgtactgtgtgcgaggataccgaaaggcagctgcgcgaatgtaccagatgggcagacgccgagtgcgaggaaatcccagggcgatggattactcggtccaccccccctgaaggatcagacagcaccgcaccatctacacaggagccagaagcaccaccagagcaggatctgatcgcctccaccgtggctggcgtggtcacaactgtcatggggagctctcagccagtggtcacccggggcaccacagataacctgattcccgtgtattgctccatcctggcagccgtcgtcgtcggactggtggcatacatcgccttcaagcggtgactcgagacctagaaaaacatggagcaatcacaagtagcaatacagcagctaacaatgctgcttgtgcctggctagaagcacaagaggaggaagaggtgggttttccagtcacacctcaggtacctttaagaccaatgacttacaaggcagctgtagatcttagccactttttaaaagaaaaggggggactggaagggctaattcactcccaaagaagacaagatatccttgatctgtggatctaccacacacaaggctacttccctgattggcagaactacacaccagggccaggggtcagatatccactgacctttggatggtgctacaagctagtaccagttgagccagataaggtagaagaggccaataaaggagagaacaccagcttgttacaccctgtgagcctgcatggaatggatgaccctgagagagaagtgttagagtggaggtttgacagccgcctagcatttcatcacgtggcccgagagctgcatccggagtacttcaagaactgctgacatcgagcttgctacaagggactttccgctggggactttccagggaggcgtggcctgggcgggactggggagtggcgagccctcagatgctgcatataagcagctgctttttgcctgtactgggtctctctggttagaccagatctgagcctgggagctctctggctaactagggaacccactgcttaagcctcaataaagcttgccttgagtgcttcaagtagtgtgtgcccgtctgttgtgtgactctggtaactagagatccctcagacccttttagtcagtgtggaaaatctctagcacccaggaggtagaggttgcagtgagccaagatcgcgccactgcattccagcctgggcaagaaaacaagactgtctaaaataataataataagttaagggtattaaatatatttatacatggaggtcataaaaatatatatatttgggctgggcgcagtggctcacacctgcgcccggccctttgggaggccgaggcaggtggatcacctgagtttgggagttccagaccagcctgaccaacatggagaaaccccttctctgtgtatttttagtagattttattttatgtgtattttattcacaggtatttctggaaaactgaaactgtttttcctctactctgataccacaagaatcatcagcacagaggaagacttctgtgatcaaatgtggtgggagagggaggttttcaccagcacatgagcagtcagttctgccgcagactcggcgggtgtccttcggttcagttccaacaccgcctgcctggagagaggtcagaccacagggtgagggctcagtccccaagacataaacacccaagacataaacacccaacaggtccaccccgcctgctgcccaggcagagccgattcaccaagacgggaattaggatagagaaagagtaagtcacacagagccggctgtgcgggagaacggagttctattatgactcaaatcagtctccccaagcattcggggatcagagtttttaaggataacttagtgtgtagggggccagtgagttggagatgaaagcgtagggagtcgaaggtgtccttttgcgccgagtcagttcctgggtgggggccacaagatcggatgagccagtttatcaatccgggggtgccagctgatccatggagtgcagggtctgcaaaatatctcaagcactgattgatcttaggttttacaatagtgatgttaccccaggaacaatttggggaaggtcagaatcttgtagcctgtagctgcatgactcctaaaccataatttcttttttgtttttttttttttatttttgagacagggtctcactctgtcacctaggctggagtgcagtggtgcaatcacagctcactgcagcctcaacgtcgtaagctcaagcgatcctcccacctcagcctgcctggtagctgagactacaagcgacgccccagttaatttttgtatttttggtagaggcagcgttttgccgtgtggccctggctggtctcgaactcctgggctcaagtgatccagcctcagcctcccaaagtgctgggacaaccggggccagtcactgcacctggccctaaaccataatttctaatcttttggctaatttgttagtcctacaaaggcagtctagtccccaggcaaaaagggggtttgtttcgggaaagggctgttactgtctttgtttcaaactataaactaagttcctcctaaacttagttcggcctacacccaggaatgaacaaggagagcttggaggttagaagcacgatggaattggttaggtcagatctctttcactgtctgagttataattttgcaatggtggttcaaagactgcccgcttctgacaccagtcgctgcattaatgaatcggccaacgcgcggggagaggcggtttgcgtattgggcgctcttccgcttcctcgctcactgactcgctgcgctcggtcgttcggctgcggcgagcggtatcagctcactcaaaggcggtaatacggttatccacagaatcaggggataacgcaggaaagaacatgtgagcaaaaggccagcaaaaggccaggaaccgtaaaaaggccgcgttgctggcgtttttccataggctccgcccccctgacgagcatcacaaaaatcgacgctcaagtcagaggtggcgaaacccgacaggactataaagataccaggcgtttccccctggaagctccctcgtgcgctctcctgttccgaccctgccgcttaccggatacctgtccgcctttctcccttcgggaagcgtggcgctttctcatagctcacgctgtaggtatctcagttcggtgtaggtcgttcgctccaagctgggctgtgtgcacgaaccccccgttcagcccgaccgctgcgccttatccggtaactatcgtcttgagtccaacccggtaagacacgacttatcgccactggcagcagccactggtaacaggattagcagagcgaggtatgtaggcggtgctacagagttcttgaagtggtggcctaactacggctacactagaagaacagtatttggtatctgcgctctgctgaagccagttaccttcggaaaaagagttggtagctcttgatccggcaaacaaaccaccgctggtagcggtggtttttttgtttgcaagcagcagattacgcgcagaaaaaaaggatctcaagaagatcctttgatcttttctacggggtctgacgctcagtggaacgaaaactcacgttaagggattttggtcatgagattatcaaaaaggatcttcacctagatccttttaaattaaaaatgaagttttaaatcaatctaaagtatatatgagtaaacttggtctgacagttaccaatgcttaatcagtgaggcacctatctcagcgatctgtctatttcgttcatccatagttgcctgactccccgtcgtgtagataactacgatacgggagggcttaccatctggccccagtgctgcaatgataccgcgagacccacgctcaccggctccagatttatcagcaataaaccagccagccggaagggccgagcgcagaagtggtcctgcaactttatccgcctccatccagtctattaattgttgccgggaagctagagtaagtagttcgccagttaatagtttgcgcaacgttgttgccattgctacaggcatcgtggtgtcacgctcgtcgtttggtatggcttcattcagctccggttcccaacgatcaaggcgagttacatgatcccccatgttgtgcaaaaaagcggttagctccttcggtcctccgatcgttgtcagaagtaagttggccgcagtgttatcactcatggttatggcagcactgcataattctcttactgtcatgccatccgtaagatgcttttctgtgactggtgagtactcaaccaagtcattctgagaatagtgtatgcggcgaccgagttgctcttgcccggcgtcaatacgggataataccgcgccacatagcagaactttaaaagtgctcatcattggaaaacgttcttcggggcgaaaactctcaaggatcttaccgctgttgagatccagttcgatgtaacccactcgtgcacccaactgatcttcagcatcttttactttcaccagcgtttctgggtgagcaaaaacaggaaggcaaaatgccgcaaaaaagggaataagggcgacacggaaatgttgaatactcatactcttcctttttcaatattattgaagcatttatcagggttattgtctcatgagcggatacatatttgaatgtatttagaaaaataaacaaataggggttccgcgcacatttccccgaaaagtgccacctgacgtctaagaaaccattattatcatgacattaacctataaaaataggcgtatcacgaggccctttcgtctcgcgcgtttcggtgatgacggtgaaaacctctgacacatgcagctcccggagacggtcacagcttgtctgtaagcggatgccgggagcagacaagcccgtcagggcgcgtcagcgggtgttggcgggtgtcggggctggcttaactatgcggcatcagagcagattgtactgagagtgcaccatatgcggtgtgaaataccgcacagatgcgtaaggagaaaataccgcatcaggcgccattcgccattcaggctgcgcaactgttgggaagggcgatcggtgcgggcctcttcgctattacgccaggggaggcagagattgcagtaagctgagatcgcagcactgcactccagcctgggcgacagagtaagactctgtctcaaaaataaaataaataaatcaatcagatattccaatcttttcctttatttatttatttattttctattttggaaacacagtccttccttattccagaattacacatatattctatttttctttatatgctccagttttttttagaccttcacctgaaatgtgtgtatacaaaatctaggccagtccagcagagcctaaaggtaaaaaataaaataataaaaaataaataaaatctagctcactccttcacatcaaaatggagatacagctgttagcattaaataccaaataacccatcttgtcctcaataattttaagcgcctctctccaccacatctaactcctgtcaaaggcatgtgccccttccgggcgctctgctgtgctgccaaccaactggcatgtggactctgcagggtccctaactgccaagccccacagtgtgccctgaggctgccccttccttctagcggctgcccccactcggctttgctttccctagtttcagttacttgcgttcagccaaggtctgaaactaggtgcgcacagagcggtaagactgcgagagaaagagaccagctttacagggggtttatcacagtgcaccctgacagtcgtcagcctcacagggggtttatcacattgcaccctgacagtcgtcagcctcacagggggtttatcacagtgcacccttacaatcattccatttgattcacaatttttttagtctctactgtgcctaacttgtaagttaaatttgatcagaggtgtgttcccagaggggaaaacagtatatacagggttcagtactatcgcatttcaggcctccacctgggtcttggaatgtgtcccccgaggggtgatgactacctcagttggatctccacaggtcacagtgacacaagataaccaagacacctcccaaggctaccacaatgggccgccctccacgtgcacatggccggaggaactgccatgtcggaggtgcaagcacacctgcgcatcagagtccttggtgtggagggagggaccagcgcagcttccagccatccacctgatgaacagaacctagggaaagccccagttctacttacaccaggaaaggc
